# Secretome from In Vitro Mechanically Loaded Myoblasts Induces Tenocyte Migration, Transition to a Fibroblastic Phenotype and Suppression of Collagen Production

**DOI:** 10.3390/ijms222313089

**Published:** 2021-12-03

**Authors:** Xin Zhou, Junhong Li, Antonios Giannopoulos, Paul J. Kingham, Ludvig J. Backman

**Affiliations:** 1 Department of Integrative Medical Biology (IMB), Faculty of Medicine, Umeå University, 90187 Umeå, Sweden; xin.zhou@umu.se (X.Z.); junhong.li@umu.se (J.L.); antonios.giannopoulos@umu.se (A.G.); paul.kingham@umu.se (P.J.K.); 2 Section of Physiotherapy, Department of Community Medicine and Rehabilitation, Faculty of Medicine, Umeå University, 90187 Umeå, Sweden

**Keywords:** collagen, differentiation, mechanical loading, migration, myoblast, proliferation, secretome, tenocyte

## Abstract

It is known that mechanical loading of muscles increases the strength of healing tendon tissue, but the mechanism involved remains elusive. We hypothesized that the secretome from myoblasts in co-culture with tenocytes affects tenocyte migration, cell phenotype, and collagen (Col) production and that the effect is dependent on different types of mechanical loading of myoblasts. To test this, we used an in vitro indirect transwell co-culture system. Myoblasts were mechanically loaded using the FlexCell^®^ Tension system. Tenocyte cell migration, proliferation, apoptosis, collagen production, and several tenocyte markers were measured. The secretome from myoblasts decreased the Col I/III ratio and increased the expression of tenocyte specific markers as compared with tenocytes cultured alone. The secretome from statically loaded myoblasts significantly enhanced tenocyte migration and Col I/III ratio as compared with dynamic loading and controls. In addition, the secretome from statically loaded myoblasts induced tenocytes towards a myofibroblast-like phenotype. Taken together, these results demonstrate that the secretome from statically loaded myoblasts has a profound influence on tenocytes, affecting parameters that are related to the tendon healing process.

## 1. Introduction

Mechanical loading is well known to improve tendon healing [1,2,3,4]. A healing tendon that is loaded becomes several-fold stronger, but the mechanism is not fully understood. It is thought that the effect of loading during tendon healing is exerted through mechanotransduction, i.e., that cells detect mechanical loading and transform this into a cellular response [5,6,7,8]. Another theory is that mechanical loading might induce microdamage of healing tissue which triggers inflammation [9]. Notably, both of these theories focus on local events within the tendon. Paracrine influence from tissue surrounding the tendon might also contribute to tendon healing. For example, cells in the paratenon, which surround the tendon, contribute to tendon healing [10]. Anatomically, the tendon is a strong dense tissue that connects muscle to bone. While muscle–bone crosstalk has been extensively studied (reviewed by [11,12,13]), muscle–tendon crosstalk has so far been largely disregarded.

Skeletal muscle is an active endocrine organ that communicates in an auto-, or paracrine manner via secretion of proteins and other molecules, the so-called secretome [14,15,16]. The secretome cross-talk between cells induces a multitude of functions such as cell proliferation, migration, collagen production, and cell differentiation [17,18,19,20]. More specifically, it has been shown that conditioned media derived from muscle tissue accelerates femoral tunnel closure in anterior cruciate ligament reconstruction in vivo [21]. Moreover, covering an open bone fracture with a muscle flap leads to accelerated healing and reduced levels of deep infection [22]. The secretome from mechanically loaded muscle cells has been shown to contain increased levels of growth factors, such as fibroblast growth factor (FGF) [23], which can potentially bind to receptors expressed by tendon cells to improve the healing process [24]. Collectively, these findings suggest that muscle secretome might improve tendon healing.

Clinical variations of mechanical loading are known to have different impact on tendon healing. Isolated mild eccentric training stimulates healing of degenerative tendons while mild concentric training exaggerates the injury [25]. The type of loading regime is also known to impact the content of cytokines in the blood plasma. Static loading increases the concentration of interleukin 15 (IL-15) while dynamic does not [26]. Moreover, the intensity of loading has an impact, i.e., in moderate-intensity resistance exercise, the serum level of insulin-like growth factor (IGF-1) was found to be significantly increased as compared to with high-intensity conditions [27]. Mechanical stretching is known to modulate IGF-1 secretion in muscle cells [28,29], thus the secreted factors from muscle influence the composition of molecules in the circulating blood. IGF-1 signaling is suggested to be required for correct human postnatal tendon growth [30]. However, whether different loading regimes could affect the muscle cell secretome, and thus lead to different outcomes in tendon healing is unknown.

The tendon healing process consists of three overlapping phases: inflammatory, proliferative, and remodelling. Immune cells are first recruited into the injury site during the inflammatory phase and orchestrate the healing process. Subsequently, tenocytes migrate to the injury site and start to proliferate and produce extracellular matrix components such as collagens (proliferation phase) [31,32,33]. Migration of tendon cells to the injury site and subsequent proliferation are crucial steps of the tendon healing process. Chen et al. found that secreted factors from rat bone marrow mesenchymal stem cells enhance tenocyte migration and proliferation [34]. This finding suggests that tenocytes are responsive to secreted factors from other closely related cells. The secretome of skeletal muscle cells contains many growth factors and cytokines that are related to migration and proliferation [16,35].

Later in the remodelling phase, there is a denser connective tissue dominated by collagen I (Col I) produced by the tenocytes and a lower amount of collagen III (Col III). The ratio of Col I/III is therefore used to analyze the collagen composition during different phases of tendon healing. The production of collagen is closely related to the cell phenotype [32,36]. As an example, higher scleraxis (Scx) expression (a tenocyte phenotype marker), is related to higher Col I production [37]. Thus, tenocytes control the production, as well as organization and maintenance of the extracellular matrix (ECM) of tendons [38]. There are many markers characteristic of tenocyte differentiation. Tenascin-C (Tnc) and vimentin (Vim) as well as Scx are markers that are commonly used to identify tenocytes [39,40]. S100 calcium binding protein A4 (S100a4) and Actin alpha 2, smooth muscle (Acta2, also termed α-SMA) are two markers associated with tendon healing. It has been demonstrated that fibroblasts expressing S100a4 are positively correlated with fibrotic tendon healing [41,42]. Furthermore, tendon cells exhibiting the ultrastructural features of smooth muscle cells express the Acta2 gene during tendon healing [43,44]. CD146 (Mcam) is a marker of tendon stem/progenitor cells [45]. Thus, analysis of the col I/III ratio and the phenotypic markers Scx, Tnc, Vim, Acta2, and S100a4 facilitates the understanding of tendon healing.

We hypothesized that (1) the secretome from myoblasts affects tenocytes and the parameters associated with tendon healing and that (2) different mechanical loading regimes of myoblasts induce different responses in tenocytes. In this study, we have investigated the parameters associated with tendon healing including (i) migration, (ii) proliferation (iii) cell phenotype, and (iv) collagen production. To overcome the complex in vivo system, we tested our hypothesis by using an in vitro indirect co-culture system using transwell inserts, culturing tenocytes in the presence of myoblasts. Myoblasts were mechanically loaded using the FlexCell^®^ system.

## 2. Results

### 2.1. Secretome from Statically Loaded Myoblasts Enhances Tenocyte Migration

**Figure 1:**
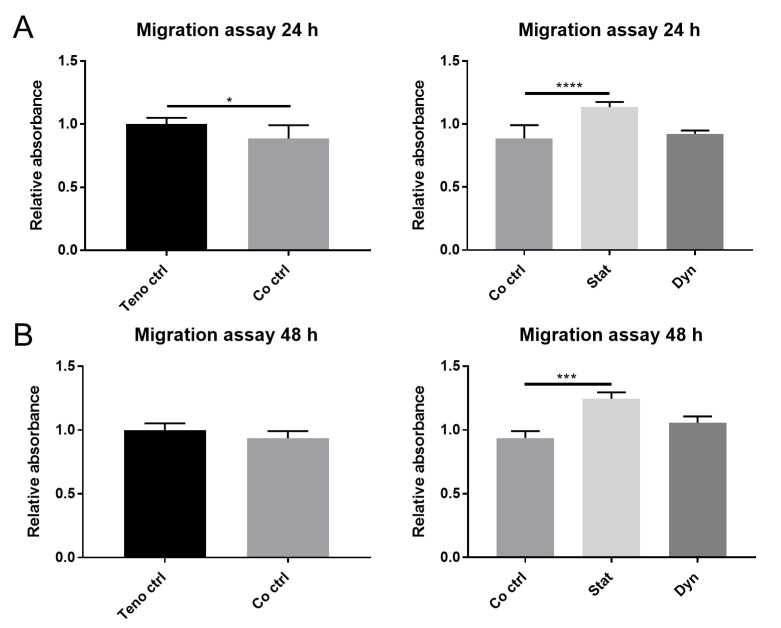
Cell migration of tenocytes co-cultured with/without mechanically loaded myoblasts. (**A**,**B**) Tenocyte migration was measured by transwell assay. Migrated cells were quantified by crystal violet staining. Teno ctrl: tenocytes alone, Co ctrl: tenocytes co-cultured with myoblasts, Dyn: tenocyte co-cultured with dynamically loaded myoblasts, Stat: tenocyte co-cultured with statically loaded myoblasts. Data are represented as mean ± standard deviation. n = 3. Student’s *t*-test was performed when comparing Teno ctrl and co ctrl. One-way ANOVA with Tukey’s multiple comparisons was performed when comparing between Co ctrl, Dyn and Stat groups. * *p* < 0.05, *** *p* < 0.001, **** *p* < 0.0001.

We first examined the effect of myoblast secretome on tenocyte migration in co-culture with mechanically unloaded myoblasts using a transwell assay. Myoblasts in co-culture with tenocytes significantly decreased the migration of tenocytes after 24 h (Figure 1A left panel) as compared with tenocytes alone; however, this effect was not seen at 48 h (Figure 1B left panel). Next, we tested whether static and dynamic loading of myoblasts could affect tenocyte migration. Dynamically loaded myoblasts did not affect tenocyte migration at 24 h or 48 h compared with unloaded myoblasts. However, statically loaded myoblasts significantly enhanced tenocyte migration at both 24 h and 48 h compared with unloaded myoblasts (Figure 1A,B right panel). The data suggest that there are differential responses in tenocyte migration capacity, relative to the type of loading on myoblasts. Furthermore, the secretome from statically loaded myoblasts does induce the migration of tenocytes.

### 2.2. Mechanically Loaded Myoblasts Have No Effects on Tenocyte Proliferation and Apoptosis

**Figure 2:**
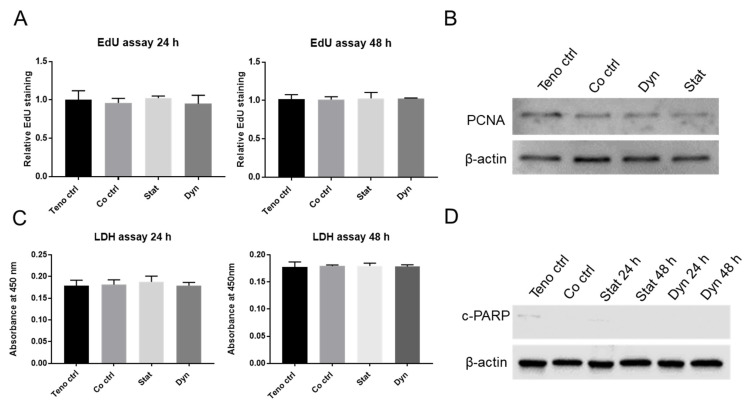
Cell proliferation and apoptosis of tenocytes co-cultured with/without mechanically loaded myoblasts. (**A**) Relative EdU counting in tenocytes co-cultured with mechanically loaded myoblasts. (**B**) Western blot images of PCNA in tenocytes after 48 h. (**C**) Quantification of LDH content in the medium. (**D**) Western blot images of c-PARP. Teno ctrl: tenocytes alone, Co ctrl: tenocytes co-cultured with myoblasts, Dyn: tenocyte co-cultured with dynamically loaded myoblasts, Stat: tenocyte co-cultured with statically loaded myoblasts. Data are represented as mean ± standard deviation. n = 3. One-way ANOVA with Tukey’s multiple comparisons was performed when comparing between Teno ctrl, Co ctrl, Dyn and Stat groups in (**A**,**C**).

Cell turnover is, together with migration, an important aspect of tendon healing. We used EdU staining to quantify the proliferation rate of tenocytes. No significant difference in proliferation was detected among all groups at 24 h and 48 h (Figure 2A). In addition, no major differences of the proliferative marker Proliferating Cell Nuclear Antigen (PCNA) was found on Western blots at 48 h (Figure 2B). Cell death is another factor that influences tenocyte turnover. We used the Lactate Dehydrogenase (LDH) Assay to quantify cell death in tenocytes. No significant difference of LDH levels was found among all groups at 24 h and 48 h, indicating no induction of either apoptosis and/or necrosis of tenocytes (Figure 2C). This result was further confirmed by Western blot which showed no major differences of cleaved-Poly(ADP-ribose) polymerase (c-PARP), a marker of late apoptotic activity (Figure 2D). Overall, these results suggest that the secretome from mechanically loaded or unloaded myoblasts does not affect tenocyte cell turnover.

### 2.3. Secretome from Mechanically Loaded Myoblasts Alters Collagen Expression of Tenocytes

**Figure 3:**
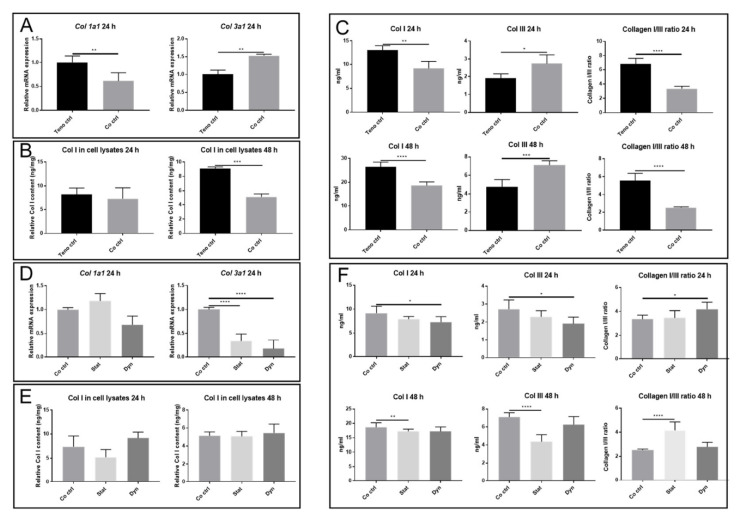

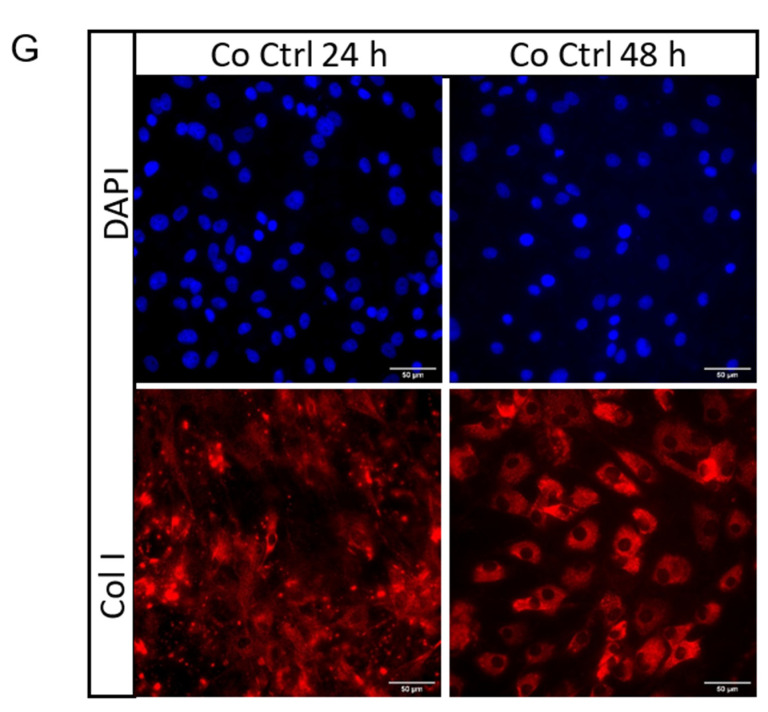
Col I/III production from co-cultures with and without mechanically loaded myoblasts. (**A**,**D**) Relative mRNA expression of *Col 1a1* and *Col 3a1* in tenocytes. (**B**,**E**) Quantification of Col 1 content in tenocyte lysate by ELISA. (**C**,**F**) Quantification of Col I/III content and the ratio in the cell culture medium. (**G**) Immunofluorescence staining of Col I on tenocytes. Teno ctrl: tenocytes alone, Co ctrl: tenocytes co-cultured with myoblasts, Dyn: tenocyte co-cultured with dynamically loaded myoblasts, Stat: tenocyte co-cultured with statically loaded myoblasts. Data are represented as mean ± standard deviation. n = 3. Student’s *t*-test was performed when comparing Teno ctrl and co ctrl. One-way ANOVA with Tukey’s multiple comparisons was performed when comparing between Co ctrl, Dyn and Stat groups. * *p* < 0.05, ** *p* < 0.01, *** *p* < 0.001, **** *p* < 0.0001.

To examine whether unloaded or mechanically loaded myoblasts affect tenocyte collagen expression, we measured *Col 1a1* and *Col 3a1* at mRNA level and the concentration of Col I and Col III within the cell (cell lysate), as well as that secreted into the medium. Secretome from unloaded myoblasts suppressed the mRNA expression of *Col 1a1* in tenocytes as compared with tenocytes alone. In contrast, *Col 3a1* mRNA expression was increased in tenocytes co-cultured with unloaded myoblasts (Figure 3A). Col I levels within the cells were reduced in tenocytes co-cultured with unloaded myoblasts at 48 h, as measured by ELISA (Figure 3B). Col III within cells was not detectable by the ELISA kit we used. The levels of Col I secretion were reduced and Col III secretion was increased in the medium from tenocytes co-cultured with unloaded myoblasts (Figure 3C). Additionally, immunofluorescence staining demonstrated that there was a reduction of Col I deposition outside of tenocytes co-cultured with unloaded myoblasts at 48 h as compared with 24 h, suggesting that Col I was released into the medium at 48 h. This might contribute to the general increase of Col I content at 48 h as compared with 24 h (Figure 3G). These results demonstrate that the secretome from unloaded myoblasts regulates the Col I/III ratio in tenocytes at both the mRNA and protein secretion levels.

We were also interested in determining whether different mechanical loading protocols could affect collagen production in the co-culture system. Neither static nor dynamic loading of myoblasts changed *Col 1a1* mRNA expression in the tenocytes at 24 h (Figure 3D). In contrast, *Col 3a1* mRNA expression in tenocytes was significantly reduced following co-culture with both dynamically and statically loaded myoblasts (Figure 3D). Secretome from mechanically loaded myoblasts did not change Col I content within the cells (Figure 3E). The concentrations of Col I and Col III in the medium at 24 h were significantly decreased in the dynamically loaded myoblasts group as compared with the unloaded myoblast group (Col I; 7.29 ± 0.98 ng/mL vs. 9.15 ± 1.25 ng/mL, *p* < 0.05. Col III; 2.29 ± 0.24 ng/mL vs. 2.72 ± 0.36 ng/mL, *p* < 0.05) (Figure 3F upper panel). The concentrations of Col I and Col III in the medium at 48 h were significantly decreased in the statically loaded myoblasts group as compared with the unloaded myoblast group (Col I; 17.20 ± 0.75 ng/mL vs. 18.64 ± 1.44 ng/mL in control, *p* < 0.01. Col III; 4.46 ± 0.55 ng/mL vs. 7.11 ± 0.33 ng/mL in control, *p* < 0.0001) (Figure 3F lower panel). Overall, these results indicate that the myoblast secretome alters the concentration of Col I and Col III in the medium and mechanical loading of myoblasts changes the collagen production in tenocytes.

### 2.4. Statically Loaded Myoblasts Change the Expression of Tenocyte Markers

**Figure 4:**
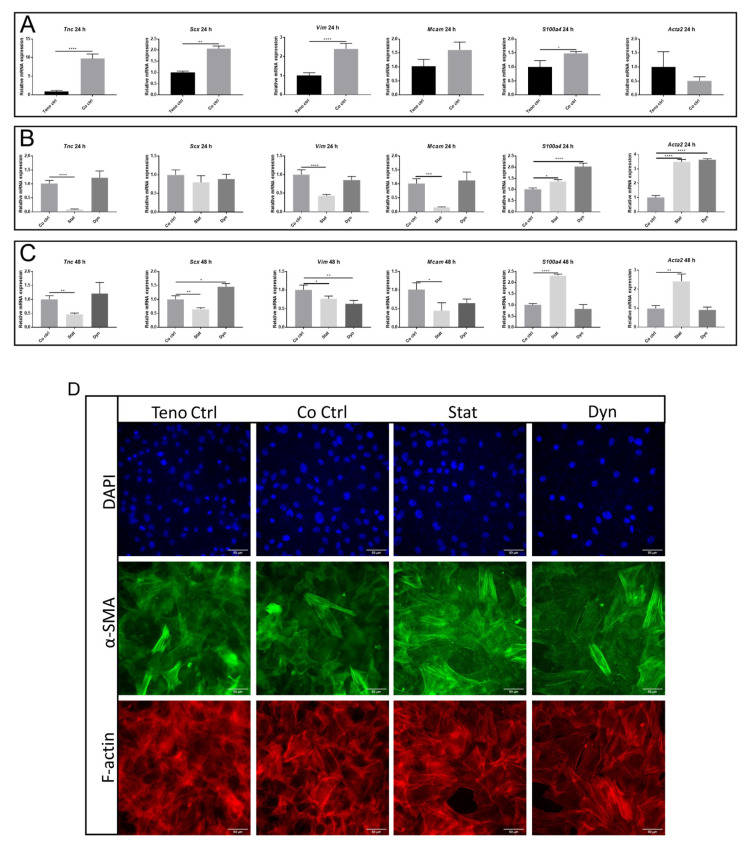
Expression of tenocyte markers in tenocytes co-cultured with or without mechanically loaded myoblasts. (**A**) mRNA expression of tenocyte markers in tenocytes co-cultured with unloaded myoblasts and tenocytes alone at 24 h. mRNA expression of tenocyte markers in tenocytes co-cultured with unloaded or mechanically loaded myoblasts at 24 h (**B**) and 48 h (**C**). (**D**) Immunofluorescence staining was performed to analyze the expression of α-SMA and F-actin between groups after 48 h of culture. *Tnc*: Tenascin-C; *Scx*: Scleraxis; *Vim*: Vimentin; *Acta2*: Actin alpha 2, smooth muscle; *S100a4*: S100 calcium-binding protein A4; *Mcam*: melanoma cell adhesion molecule. Teno ctrl: tenocytes alone, Co ctrl: tenocytes co-cultured with myoblasts, Dyn: tenocyte co-cultured with dynamically loaded myoblasts, Stat: tenocyte co-cultured with statically loaded myoblasts. Data are represented as mean ± standard deviation. n = 3. Student’s *t*-test was performed when comparing Teno ctrl and co ctrl. One-way ANOVA with Tukey’s multiple comparisons was performed when comparing between Co ctrl, Dyn and Stat groups. * *p* < 0.05, ** *p* < 0.01, *** *p* < 0.001, **** *p* < 0.0001.

Tenogenic differentiation is an important aspect of tendon composition. We observed an increase of *Tnc* mRNA level in tenocytes in co-culture with the unloaded myoblasts group compared with the tenocytes group (9.70 ± 1.00 fold vs. teno ctrl, *p* < 0.001), while *S100a4, Scx,* and *Vim* showed a moderate increase at mRNA level in tenocytes in co-culture with unloaded myoblasts compared with tenocytes alone at 24 h (1.48 ± 0.07, 2.06 ± 0.13 and 2.39 ± 0.25 fold vs. teno ctrl, respectively, *p* < 0.05). *Acta2* and *Mcam* showed no significant changes (Figure 4A).

The expression of all three markers of tenogenic differentiation (*Scx, Vim,* and *Tnc*) were significantly reduced in tenocytes in co-culture with statically loaded myoblasts as compared with unloaded myoblasts. Secretome from statically loaded myoblasts increased the expression of *S100a4* and *Acta2* in tenocytes as compared with unloaded myoblasts at 24 h and 48 h (Figure 4B,C). In addition, *Mcam* was decreased in tenocytes after co-culture with statically loaded myoblasts at 24 h and 48 h. Secretome from dynamically loaded myoblasts also induced upregulation of *S100a4* and *Acta2* in tenocytes at 24 h, but did not affect tenocyte markers (Figure 4B). There were increased *Scx* levels and decreased *Vim* expression in tenocytes after co-culture with dynamically loaded myoblasts at 48 h, but *S100a4* and *Acta2* expression remained at the control level (Figure 4C). *Mcam* did not show a significant change in expression in tenocytes after co-culture with dynamically loaded myoblasts at 24 h and 48 h. Additionally, a more pronounced α-SMA expression was detected in tenocytes co-cultured with statically loaded myoblasts as compared to dynamically loaded myoblasts, tenocytes co-cultured with unloaded myoblasts, and tenocytes alone after 48 h, as measured by immunofluorescence (Figure 4D). Taken together, these results showed that secretome from dynamically loaded myoblasts did not change the tenocyte differentiation to the same extent as statically loaded myoblasts.

## 3. Discussion

While the theories of mechanotransduction and microdamage have been put forward to explain how tendon healing is accomplished [46,47], in this study, we have explored the possibility that parameters of tendon healing may be affected by the secretome from mechanically loaded muscles. By using indirect co-culture of myoblasts with tenocytes, we demonstrated that the ratio of Col I/III was decreased and that tenocytes had higher expression of tenocyte markers. By applying different mechanical loading regimes on myoblasts in co-culture with unloaded tenocytes, we showed that statically loaded myoblasts induced an increased tenocyte migration, increased the ratio of Col I/III, and induced a tenocyte differentiation towards myofibroblast-like cells as compared with dynamically loaded myoblasts. This is, to our knowledge, the first in vitro study to evaluate the effect of myoblast-derived secretome on tenocytes and to evaluate how this is affected by mechanically loading the cells.

The purpose of the loading regime applied in this study was to stimulate the mechanical response of myoblasts, but not to inflict irreversible damage on cells. Baccam et al. used a similar loading protocol (0.5 Hz, 6% stretch, 3 h rest) to enhance follistratin secretion in myotubes [48]. The 5% strain used is considered a moderate mechanical stress on myoblasts, as >10% strain leads to increased apoptosis and up-regulation of the pro-apoptotic factors in muscle cells [49,50,51]. Insertion of rest periods between cycles of mechanical stimulation is usually incorporated to allow cells to recover, although the duration varies between studies [23,48]. The morphology of the myoblasts was analysed under the microscope, before and after loading, and the cells showed no obvious changes in morphology (data not shown). Taking this into account, in combination with the data obtained from tenocytes, we consider that the loading regime used in this study was not deleterious to myoblasts.

Studies have shown that the cell secretome can promote migration and proliferation of various cell types [18,52,53]. Interestingly, we found that only static loading of myoblasts induced tenocyte migration, while dynamic loading had no effects. This significantly enhanced migration on tenocytes is not due to increased cell turnover since we did not detect any changes in proliferation or apoptosis. Cell proliferation and migration are reciprocally controlled by the concentration of soluble cytokines sensed by the cells [54]. For example, platelet-derived growth factor (PDGF), which is found in the myoblast secretome, stimulates migration but not proliferation of osteoblastic cells at concentrations ranging from 5–50 ng/mL [55]. Another study showed that PDGF induced a migration response at concentrations of 1–5 ng/mL, while at higher concentrations (>5 ng/mL), PDGF induced proliferation but not migration of NIH3T3 fibroblasts [56]. Additionally, Toti et al. showed that microvesicles-mediated transfer of Galectin-1 proteins from fibroblasts stimulates cell migration [57]. These studies suggest that the concentration of cytokines as well as specific proteins in the secretome from myoblasts lead to different outcomes on cell migration and proliferation.

A decreased Col I/III ratio has been widely used as a marker of the aging tendon [58] and increased tendon pathology [59]. Our results showed that the secretome from statically loaded myoblasts increased the Col I/III ratio, thus a loading regime that induces improved collagen composition as compared with dynamic loading. The myoblast secretome contains many interleukins (IL) (1β, 2, 4, 6, 7, 8, 10, 13, 17A, 25, 34) and tumor necrosis factor α (TNF-α) [15,60,61,62] which could explain the changed expression profile of Col I/III in tenocytes. Some of the interleukins decrease Col I production, such as IL-1 β [63], IL-7 [64], IL-10 [65], and IL-17A [66], while IL-4, IL-25, IL-34, IL-6, and TNF-α increase Col I production [67,68,69,70]. Among these factors, IL-1 and TNF-α are inflammatory cytokines that both trigger NF-kappaB signaling (NF-κB). A recent report showed that NF-κB signaling decreased the Col I/III ratio during tendon healing [71]. In addition, IL-17 has been shown to decrease the ratio of Col I/III ratio in tendons [72]. IL-17 also activates the canonical NF-κB signaling pathway [73,74]. Thus, the increased Col I/III ratio that we detected in tenocytes exposed to secretome from statically loaded myoblasts, as compared with dynamically loaded myoblasts, could be caused by altered NF-κB stimulation.

The secretome from unloaded myoblasts increased the expression of tenocyte markers (*Scx, Tnc,* and *Vim*) while statically loaded myoblasts decreased the expression. This, in combination with the fact that the secretome from mechanically loaded myoblasts results in upregulated *Acta2* expression, suggests that the secretome from statically loaded myoblasts stimulates a myofibroblastic differentiation. This is further supported by an upregulation of *S100a4*. These two markers (*Acta2* and *S100a4*) are indicative of fibrotic tendon healing. However, Ackerman et al. found that completely blocking the action of the S100a4 protein resulted in a decreased tendon fibrosis, but the tendon itself was not as strong or functional [42]. It is known that myofibroblasts of injured tendons are responsible for increased collagen deposition [71,75]. It is speculated that there is an interplay between *S100a4* and *Acta2* expression in tenocytes which modulates different phases of tendon healing. As an example, modulation of *S100a4* and *Acta2* expression occurs during the phase of restoration of matrix integrity and deposition of excess ECM [42]. The cellular localization of S100a4 is also related to the cell phenotype. For example, in keratocytes, S100a4 is localized in the cytoplasm, but in keratocytes differentiated towards myofibroblasts, it is in the nucleus [76]. Our experiments studied mRNA expression from the whole cell lysate and therefore do not distinguish the localization within the cell. However, since it is known that the secretome from skeletal muscle cells contain two major components of the extracellular matrix, decorin and biglycan [16], which affect the TGF-β signaling by competitive binding of its transducing receptors [77], it is likely that there is a translocation of S100a4 expression in the cells. Since both *S100a4* and *Acta2* expression is increased several fold in tenocytes exposed to the secretome from statically loaded myoblasts, we suggest that this plays a central role and potentially explains the increased migration and ratio of Col I/III production.

The secretome from mechanically unloaded and loaded myoblasts has various effects on migration, collagen production, and cell characteristics which suggest that different loading regimes affect the tendon healing in a phase dependent-manner. In support of this, clinical variations of mechanical loading are known to have different impacts on tendon healing [78,79,80] and also affect the serum level of certain molecules know to be involved in tendon healing such as cartilage oligomeric matrix protein (COMP) [81,82]. Thus, another interesting and clinically useful implication could be that training of muscle closely related to the injured tendon can have beneficial healing effects. Specifically, a patient prescribed immobilization following tendon injury may benefit from training other muscles, since molecules in the circulating blood, derived from the active muscles, are altered following exercise.

In summary, in this study, we have investigated the effects of the secretome from mechanically loaded myoblasts on tenocytes in vitro. The biological endpoints included migration, proliferation/apoptosis, Col I/III expression, and cell phenotype. Secretome from statically loaded myoblasts enhanced cell migration, increased Col I/III ratio, and induced cell characteristic changes. To our knowledge, this is the first study to investigate the effects of myoblast secretome on tenocytes and whether the possible involvement is dependent on the type of mechanical loading of myoblasts in vitro.

## 4. Materials and Methods

### 4.1. Isolation and Culture of Primary Cells

Primary tendon and muscle cells were isolated from healthy female Sprague-Dawley rats of seven to eight weeks of age (ethical approval A31-19). Tendons were washed twice with phosphate-buffered saline and cut into small pieces measuring approximately 1.0 mm^3^. The pieces were digested with 1 mg/mL collagenase type I (Cell Signaling Technology, Topsfield, MA, USA) overnight. The cell pellets were collected the next day and suspended in Dulbecco’s modified Eagle’s medium (DMEM) with GlutaMAX (Sigma-Aldrich, St. Louis, MO, USA) supplemented with 1% penicillin (Sigma-Aldrich, St. Louis, MO, USA) and 10% fetal bovine serum (FBS) (Biowest, Boca Raton, FL, USA). Rat primary myoblasts were prepared as described previously [83]. Briefly, gastrocnemius muscles were cut into small pieces measuring approximately 1.0 mm^3^ in a culture dish containing DMEM with 10% FBS and 1% penicillin–streptomycin. The culture dish was incubated in a 37 °C, 5% CO_2_ incubator, with media exchange every 2 days. When the cells reached about 80% confluence, the tissue blocks were discarded and the cells were moved to a collagen-coated dish (Thermo Fisher Scientific, Waltham, MA, USA, code: A11428-01) for 15 min. This step was repeated 2 more times to eliminate the rapidly adhering cells, predominantly fibroblasts. The resulting media containing primary myoblasts were then transferred to a new flask for further culturing. Both cell types were maintained at a sub-confluent level (<80%). During experiments the FBS concentration was reduced to 1%.

### 4.2. Mechanical Strain

Myoblasts were seeded on the elastic membrane of the Bioflex 6-well plate coated with Collagen I (Flexcell International Corporation, Burlington, NC, USA) at a cell density of 3 × 10^5^ cells per well and were left to adhere overnight. The plate was placed on a circular shaped loading post of 25 mm in diameter. In the FlexCell FX-5000 tension system (Flexcell International Corporation, Burlington, NC, USA), the membrane was pulled downwards by vacuum suction which caused the membrane to stretch across the loading post. The cells received either 5% equibiaxial dynamic strain at a frequency of 1 Hz or constant, statical strain based on protocols from the literature [48,50] and preliminary data. Cells were strained for 1 h followed by a rest period of 2 h. This loading was repeated for 3 times followed by a rest period of 4 h. One cycle took in total 13 h. The protocol was repeated for 24 h or 48 h.

### 4.3. Indirect Co-Culture

Primary myoblasts were seeded in Bioflex 6-well plates at a density of 3 × 10^5^ cells per well. In parallel, tenocytes with a cell density of 3 × 10^5^ cells were added in PET transwell membrane inserts (Corning, NY, USA) with a pore size of 1.0 or 8.0 μm and kept in a separate plate in the incubator until the experiment was begun. During all experiments, the inserts were placed in indirect co-culture with the myoblasts.

### 4.4. Migration Assays

Tenocytes were added to inserts with a pore size of 8.0 μm (Corning, NY, USA) in the co-culturing system. After 24 h and 48 h, the cells that had passed through the membrane and remained attached to the bottom of the membrane were stained using 0.1% crystal violet (Sigma-Aldrich, St. Louis, MO, USA) for 30 min. Non-migratory cells remaining on the upper surface of the insert membrane were removed using cotton-tipped swabs. Finally, the inserts were placed in an extraction solution containing 30% methanol, 10% acetic acid, and 60% milli-q water before analyses at OD 590 nm in a plate reader.

### 4.5. Cell proliferative Assay

Proliferating cells were evaluated by using the Click-iT EdU Alexa Fluor 594 Imaging kit (Invitrogen, Carlsbad, CA, USA) according to the manufacturer’s instructions. Briefly, cells were incubated with 10 μM EdU for 24 and 48 h at 37 °C in the co-culture system, as described in Section 4.3. Subsequently, tenocytes in the PET transwell membrane inserts were washed twice with PBS containing 3% BSA and fixed with 3.7% formaldehyde for 15 min, before treatment with 0.5% Triton X-100 (Sigma-Aldrich, St. Louis, MO, USA) for 15 min at room temperature. The cells were then exposed to Click-iT reaction cocktail for 30 min after washing twice with PBS containing 3% BSA. Images were acquired and analyzed by Incucyte Live Cell analysis System (Essen Instruments, Ann Arbor, MI, USA). A single culture of tenocytes and a co-culture control were included in each group. Foci counting was normalized to single culture counting in each group to avoid batch-to-batch variation between groups.

### 4.6. Lactate Dehydrogenase Activity Assay

Lactate dehydrogenase (LDH) Colorimetric Assay kit (Abcam, Cambridge, UK) was performed on cell culture medium according to the manufacturer’s instructions. Briefly, culture medium was collected and centrifuged at 2000× *g* at 4 °C for 3 min to remove cell debris. Subsequently, the medium was stored at −80 °C until all groups were collected. After thawing, 50 µL of medium was mixed with 50 µL reaction mix provided by the kit. The samples were measured at OD 450 nm in a platereader after 60 min incubation at 37 °C protected from light.

### 4.7. RNA Extraction and qRT-PCR

Extraction of mRNA was performed using the RNA extraction kit (Qiagen, Venlo, The Netherlands, # 74106) according to the manufacturer’s instructions. Subsequently, a high-capacity cDNA reverse transcription kit (Thermo Fisher, Waltham, MA, USA) was used to reverse transcribe 1000 ng of RNA into cDNA. To determine the gene expression, TaqMan Gene Expression Assays (Applied Biosystems, Carlsbad, CA, USA) were used. cDNA was run in triplicates with the ViiA7 Real-Time PCR system and analyzed with the associated software (Applied Biosystems, Carlsbad, CA, USA). Gene expression was measured by using TaqMan Gene Expression Assay (Applied Biosystems, Carlsbad, CA, USA) and calculated by 2^−∆∆Ct^ method. A summary of all probes used for real-time PCR (Applied Biosystems, Carlsbad, CA, USA) is provided in Table 1.

### 4.8. Western Blot

Cells were freeze-thawed and further lysed in RIPA (radioimmunoprecipitation) lysis buffer (Thermo Fisher, Waltham, MA, USA) supplemented with protease and phosphatase inhibitor cocktail (Sigma, St. Louis, MO, USA, #P1860). Total protein concentration was determined with the Bradford assay (Bio-Rad, Hercules, CA, USA). Samples containing 20 µg of protein were separated on SDS-polyacrylamide gels and transferred to PVDF membranes (Thermo Fisher, Waltham, MA, USA). Membranes were blocked in 5% bovine serum albumin in TBS-T for 1 h before staining with primary antibodies overnight at 4 °C. After washing, the membranes were stained with HRP-conjugated secondary antibodies for 1 h at room temperature before incubation with ECL solution and then analyzed in an Odyssey Fc Dual-Mode Imaging System (LI-COR Biotechnology, Lincoln, NE, USA). A summary of all antibodies used is provided in Table 2.

### 4.9. ELISA

Medium and cell lysates were collected from each well. Briefly, the culture medium was centrifuged at 2000× *g* at 4 °C for 3 min to remove cell debris. Subsequently, the medium was stored at −80 °C until all groups were collected. Cells were freeze-thawed and further lysed in RIPA lysis buffer supplemented with protease and phosphatase inhibitor cocktail (Sigma, St. Louis, MO, USA, #P1860). Total protein concentration was determined with BCA assay (Thermo Fisher, Waltham, MA, USA). Collagen I and collagen III were assessed using Rat Collagen Type I ELISA kit (Cusabio, Wuhan, China, #CSB-E08084r) and Rat Collagen Type III ELISA kit (Cusabio, Wuhan, China, #CSB-E07924r) according to the manufacturer’s protocol. Intracellular collagen I concentration was normalized to the total protein concentration of the cell lysates.

### 4.10. Immunocytochemistry

Inserts were washed three times with PBS and cells were fixed with 10% formalin for 10 min, and then transferred into PBS at 4 °C. The samples were permeabilized with 1% Triton X-100 and blocked with 1% Bovine Serum Albumin (BSA, Sigma, St. Louis, MO, USA, #A9647). Collagen I (1:400) (Abcam, Cambridge, UK, #260043) was incubated with the samples at 4 °C overnight. After washing, fluorescein-conjugated secondary antibody Goat-anti rabbit (1:400) (Thermo Fisher, Waltham, MA, USA, #A32740) was added for 1 h at room temperature, and DAPI (Thermo Fisher, Waltham, MA, USA, #62248) was used to stain the nuclei of the cells. F-actin was stained by phalloidin (1:400) (Thermo Fisher, Waltham, MA, USA, #A22287) and α-SMA was stained by Alexa Fluor 488 conjugated antibody (1:400) (Thermo Fisher, Waltham, MA, USA, # 53976082), respectively. Detailed information on the antibodies used is summarized in Table 3.

### 4.11. Statistics

Statistical analysis was performed using Student’s *t*-test when comparing two groups. One-way ANOVA with Tukey’s multiple comparisons was performed when comparing between more than two groups. Differences were considered statistically significant at a *p*-value of <0.05. All experiments were repeated at least three times. All experimental samples were prepared in triplicates.

## Figures and Tables

**Table 1:** Probe information for real-time PCR.

Gene Symbol	Gene Name	Assay ID
*Mcam*	melanoma cell adhesion molecule, CD 146	Rn00576900_m1
*Vim*	Vimentin	Rn00579738
*Tnc*	Tenascin-C	Rn01454948
*Acta2*	actin alpha 2, smooth muscle, α-SMA	Rn01759928
*S100a4*	S100 calcium-binding protein A4	Rn01451938
*Scx*	Scleraxis bHLH transcription factor	Rn01504576
*Col1a1*	Collagen type I alpha 1 chain	Rn01463848_m1
*Col3a1*	collagen type III alpha 1 chain	Rn01437681_m1
*Rpl13a* (reference gene)	Ribosomal Protein L13a	Rn00821946_g1

**Table 2:** Antibody information for western blot.

Antibody	Company	Code	Dilution	Species	Molecular Weight (kDa)
PCNA	Cell Signaling	2586	1:1000	Mouse	36
c-PARP	Cell Signaling	9541	1:1000	Rabbit	89
β-actin	Cell Signaling	4967	1:2000	Rabbit	45
Anti-rabbit IgG, HRP-linked antibody	Cell Signaling	7074	1:2000	Goat	

**Table 3:** Antibody information for immunocytochemistry.

Antibody	Company	Code	Dilution	Species
Collagen I	Abcam	260043	1:400	Rabbit
α-SMA	Thermo Fisher	53976082	1:400	Rabbit
Fluorescein-conjugated secondary antibody Goat-anti rabbit	Thermo Fisher	A32740	1:400	Rabbit

## References

[1] Andersson T., Eliasson P., Aspenberg P. (2009). Tissue memory in healing tendons: Short loading episodes stimulate healing. J. Appl. Physiol..

[2] Eliasson P., Andersson T., Aspenberg P. (2012). Achilles tendon healing in rats is improved by intermittent mechanical loading during the inflammatory phase. J. Orthopaed. Res..

[3] Eliasson P., Andersson T., Aspenberg P. (2009). Rat Achilles tendon healing: Mechanical loading and gene expression. J. Appl. Physiol..

[4] Nourissat G., Berenbaum F., Duprez D. (2015). Tendon injury: From biology to tendon repair. Nat. Rev. Rheumatol..

[5] Wang J.H. (2006). Mechanobiology of tendon. J. Biomech..

[6] Killian M.L., Cavinatto L., Galatz L.M., Thomopoulos S. (2012). The role of mechanobiology in tendon healing. J. Shoulder Elbow Surg..

[7] Arnoczky S.P., Tian T., Lavagnino M., Gardner K. (2004). Ex vivo static tensile loading inhibits MMP-1 expression in rat tail tendon cells through a cytoskeletally based mechanotransduction mechanism. J. Orthop. Res..

[8] Wall M.E., Dyment N.A., Bodle J., Volmer J., Loboa E., Cederlund A., Fox A.M., Banes A.J. (2016). Cell Signaling in Tenocytes: Response to Load and Ligands in Health and Disease. Adv. Exp. Med. Biol..

[9] Hammerman M., Dietrich-Zagonel F., Blomgran P., Eliasson P., Aspenberg P. (2018). Different mechanisms activated by mild versus strong loading in rat Achilles tendon healing. PLoS ONE.

[10] Dyment N.A., Liu C.F., Kazemi N., Aschbacher-Smith L.E., Kenter K., Breidenbach A.P., Shearn J.T., Wylie C., Rowe D.W., Butler D.L. (2013). The Paratenon Contributes to Scleraxis-Expressing Cells during Patellar Tendon Healing. PLoS ONE.

[11] Cariati I., Bonanni R., Onorato F., Mastrogregori A., Rossi D., Iundusi R., Gasbarra E., Tancredi V., Tarantino U. (2021). Role of Physical Activity in Bone-Muscle Crosstalk: Biological Aspects and Clinical Implications. J. Funct. Morphol. Kinesiol..

[12] Bosco F., Musolino V., Gliozzi M., Nucera S., Carresi C., Zito M.C., Scarano F., Scicchitano M., Reale F., Ruga S. (2021). The muscle to bone axis (and viceversa): An encrypted language affecting tissues and organs and yet to be codified?. Pharmacol. Res..

[13] Maurel D.B., Jahn K., Lara-Castillo N. (2017). Muscle-Bone Crosstalk: Emerging Opportunities for Novel Therapeutic Approaches to Treat Musculoskeletal Pathologies. Biomedicines.

[14] Norheim F., Raastad T., Thiede B., Rustan A.C., Drevon C.A., Haugen F. (2011). Proteomic identification of secreted proteins from human skeletal muscle cells and expression in response to strength training. Am. J. Physiol. Endocrinol. Metab..

[15] Deshmukh A.S., Cox J., Jensen L.J., Meissner F., Mann M. (2015). Secretome Analysis of Lipid-Induced Insulin Resistance in Skeletal Muscle Cells by a Combined Experimental and Bioinformatics Workflow. J. Proteome Res..

[16] Henningsen J., Rigbolt K.T., Blagoev B., Pedersen B.K., Kratchmarova I. (2010). Dynamics of the skeletal muscle secretome during myoblast differentiation. Mol. Cell. Proteom..

[17] Teixeira F.G., Panchalingam K.M., Assuncao-Silva R., Serra S.C., Mendes-Pinheiro B., Patricio P., Jung S., Anjo S.I., Manadas B., Pinto L. (2016). Modulation of the Mesenchymal Stem Cell Secretome Using Computer-Controlled Bioreactors: Impact on Neuronal Cell Proliferation, Survival and Differentiation. Sci. Rep..

[18] Ledet M.M., Vasquez A.K., Rauner G., Bichoupan A.A., Moroni P., Nydam D.V., Van de Walle G.R. (2018). The secretome from bovine mammosphere-derived cells (MDC) promotes angiogenesis, epithelial cell migration, and contains factors associated with defense and immunity. Sci. Rep..

[19] Cho J.W., Kang M.C., Lee K.S. (2010). TGF-beta 1-treated ADSCs-CM promotes expression of type I collagen and MMP-1, migration of human skin fibroblasts, and wound healing in vitro and in vivo. Int. J. Mol. Med..

[20] Infante A., Rodriguez C.I. (2018). Secretome analysis of in vitro aged human mesenchymal stem cells reveals IGFBP7 as a putative factor for promoting osteogenesis. Sci. Rep..

[21] Ghebes C.A., Groen N., Cheuk Y.C., Fu S.C., Fernandes H.M., Saris D.B.F. (2018). Muscle-Secreted Factors Improve Anterior Cruciate Ligament Graft Healing: An In Vitro and In Vivo Analysis. Tissue Eng. Part A.

[22] Harry L.E., Sandison A., Paleolog E.M., Hansen U., Pearse M.F., Nanchahal J. (2008). Comparison of the healing of open tibial fractures covered with either muscle or fasciocutaneous tissue in a murine model. J. Orthop. Res..

[23] Clarke M.S., Feeback D.L. (1996). Mechanical load induces sarcoplasmic wounding and FGF release in differentiated human skeletal muscle cultures. FASEB J..

[24] Najafbeygi A., Fatemi M.J., Lebaschi A.H., Mousavi S.J., Husseini S.A., Niazi M. (2017). Effect of Basic Fibroblast Growth Factor on Achilles Tendon Healing in Rabbit. World J. Plast. Surg..

[25] Grigg N.L., Wearing S.C., Smeathers J.E. (2009). Eccentric calf muscle exercise produces a greater acute reduction in Achilles tendon thickness than concentric exercise. Br. J. Sport Med..

[26] Kapilevich L.V., Zakharova A.N., Kabachkova A.V., Kironenko T.A., Orlov S.N. (2017). Dynamic and Static Exercises Differentially Affect Plasma Cytokine Content in Elite Endurance- and Strength-Trained Athletes and Untrained Volunteers. Front. Physiol..

[27] Tsai C.L., Wang C.H., Pan C.Y., Chen F.C. (2015). The effects of long-term resistance exercise on the relationship between neurocognitive performance and GH, IGF-1, and hornocysteine levels in the elderly. Front. Behav. Neurosci..

[28] Iwanuma O., Abe S., Hiroki E., Kado S., Sakiyama K., Usami A., Ide Y. (2008). Effects of mechanical stretching on caspase and IGF-1 expression during the proliferation process of myoblasts. Zool. Sci..

[29] Perrone C.E., Fenwick-Smith D., Vandenburgh H.H. (1995). Collagen and stretch modulate autocrine secretion of insulin-like growth factor-1 and insulin-like growth factor binding proteins from differentiated skeletal muscle cells. J. Biol. Chem..

[30] Disser N.P., Sugg K.B., Talarek J.R., Sarver D.C., Rourke B.J., Mendias C.L. (2019). Insulin-like growth factor 1 signaling in tenocytes is required for adult tendon growth. FASEB J..

[31] Snedeker J.G., Foolen J. (2017). Tendon injury and repair—A perspective on the basic mechanisms of tendon disease and future clinical therapy. Acta Biomater..

[32] Thomopoulos S., Parks W.C., Rifkin D.B., Derwin K.A. (2015). Mechanisms of tendon injury and repair. J. Orthop. Res..

[33] Woo S.L., Hildebrand K., Watanabe N., Fenwick J.A., Papageorgiou C.D., Wang J.H. (1999). Tissue engineering of ligament and tendon healing. Clin. Orthop. Relat. Res..

[34] Chen Q., Liang Q., Zhuang W., Zhou J., Zhang B., Xu P., Ju Y., Morita Y., Luo Q., Song G. (2018). Tenocyte proliferation and migration promoted by rat bone marrow mesenchymal stem cell-derived conditioned medium. Biotechnol. Lett..

[35] Chan C.Y., McDermott J.C., Siu K.W. (2011). Secretome Analysis of Skeletal Myogenesis Using SILAC and Shotgun Proteomics. Int. J. Proteom..

[36] Yang G., Rothrauff B.B., Tuan R.S. (2013). Tendon and Ligament Regeneration and Repair: Clinical Relevance and Developmental Paradigm. Birth Defects Res. C.

[37] Huisman E., Lu A., McCormack R.G., Scott A. (2014). Enhanced collagen type I synthesis by human tenocytes subjected to periodic in vitro mechanical stimulation. BMC Musculoskelet. Disord..

[38] Gumucio J.P., Sugg K.B., Mendias C.L. (2015). TGF-beta Superfamily Signaling in Muscle and Tendon Adaptation to Resistance Exercise. Exerc. Sport Sci. Rev..

[39] Lee C.H., Lee F.Y., Tarafder S., Kao K., Jun Y.N., Yang G.D., Mao J.J. (2015). Harnessing endogenous stem/progenitor cells for tendon regeneration. J. Clin. Investig..

[40] Jo C.H., Lim H.J., Yoon K.S. (2019). Characterization of Tendon-Specific Markers in Various Human Tissues, Tenocytes and Mesenchymal Stem Cells. Tissue Eng. Regen. Med..

[41] Best K.T., Loiselle A.E. (2019). Scleraxis lineage cells contribute to organized bridging tissue during tendon healing and identify a subpopulation of resident tendon cells. FASEB J..

[42] Ackerman J.E., Nichols A.E.C., Studentsova V., Best K.T., Knapp E., Loiselle A.E. (2019). Cell non-autonomous functions of S100a4 drive fibrotic tendon healing. Elife.

[43] Premdas J., Tang J.B., Warner J.P., Murray M.M., Spector M. (2001). The presence of smooth muscle actin in fibroblasts in the torn human rotator cuff. J. Orthop. Res..

[44] Cadby J.A., Buehler E., Godbout C., van Weeren P.R., Snedeker J.G. (2014). Differences between the Cell Populations from the Peritenon and the Tendon Core with Regard to Their Potential Implication in Tendon Repair. PLoS ONE.

[45] Lee C., Jun Y., Kao K. (2015). Harnessing Endogenous Stem/Progenitor Cells for Tendon Regeneration. Tissue Eng. Part A.

[46] Blomgran P., Blomgran R., Ernerudh J., Aspenberg P. (2016). A possible link between loading, inflammation and healing: Immune cell populations during tendon healing in the rat. Sci. Rep..

[47] Hammerman M., Aspenberg P., Eliasson P. (2014). Microtrauma stimulates rat Achilles tendon healing via an early gene expression pattern similar to mechanical loading. J. Appl. Physiol..

[48] Baccam A., Benoni-Sviercovich A., Rocchi M., Moresi V., Seelaender M., Li Z., Adamo S., Xue Z., Coletti D. (2019). The Mechanical Stimulation of Myotubes Counteracts the Effects of Tumor-Derived Factors Through the Modulation of the Activin/Follistatin Ratio. Front. Physiol..

[49] Moustogiannis A., Philippou A., Zevolis E., Taso O., Chatzigeorgiou A., Koutsilieris M. (2020). Characterization of Optimal Strain, Frequency and Duration of Mechanical Loading on Skeletal Myotubes’ Biological Responses. In Vivo.

[50] Tan J.L., Kuang W., Jin Z.L., Jin F., Xu L., Yu Q.J., Kong L., Zeng G., Yuan X., Duan Y.Z. (2009). Inhibition of NFkappaB by activated c-Jun NH2 terminal kinase 1 acts as a switch for C2C12 cell death under excessive stretch. Apoptosis.

[51] Liu M., Huang X., Tian Y., Yan X., Wang F., Chen J., Zhang Q., Zhang Q., Yuan X. (2020). Phosphorylated GSK3beta protects stressinduced apoptosis of myoblasts via the PI3K/Akt signaling pathway. Mol. Med. Rep..

[52] Park S.R., Kim J.W., Jun H.S., Roh J.Y., Lee H.Y., Hong I.S. (2018). Stem Cell Secretome and Its Effect on Cellular Mechanisms Relevant to Wound Healing. Mol. Ther..

[53] Kamprom W., Kheolamai P., Supokawej A., Wattanapanitch M., Laowtammathron C., Roytrakul S., Issaragrisil S. (2016). Endothelial Progenitor Cell Migration-Enhancing Factors in the Secretome of Placental-Derived Mesenchymal Stem Cells. Stem Cells Int..

[54] De Donatis A., Ranaldi F., Cirri P. (2010). Reciprocal control of cell proliferation and migration. Cell Commun. Signal..

[55] Kim S.J., Kim S.Y., Kwon C.H., Kim Y.K. (2007). Differential effect of FGF and PDGF on cell proliferation and migration in osteoblastic cells. Growth Factors.

[56] De Donatis A., Comito G., Buricchi F., Vinci M.C., Parenti A., Caselli A., Camici G., Manao G., Ramponi G., Cirri P. (2008). Proliferation versus migration in platelet-derived growth factor signaling: The key role of endocytosis. J. Biol. Chem..

[57] Toti A., Santi A., Pardella E., Nesi I., Tomasini R., Mello T., Paoli P., Caselli A., Cirri P. (2021). Activated fibroblasts enhance cancer cell migration by microvesicles-mediated transfer of Galectin-1. J. Cell. Commun. Signal..

[58] Smith R.K., Birch H., Patterson-Kane J., Firth E.C., Williams L., Cherdchutham W., van Weeren W.R., Goodship A.E. (1999). Should equine athletes commence training during skeletal development?: Changes in tendon matrix associated with development, ageing, function and exercise. Equine Vet. J. Suppl..

[59] Goncalves-Neto J., Witzel S.S., Teodoro W.R., Carvalho-Junior A.E., Fernandes T.D., Yoshinari H.H. (2002). Changes in collagen matrix composition in human posterior tibial tendon dysfunction. Joint Bone Spine.

[60] Hartwig S., Raschke S., Knebel B., Scheler M., Irmler M., Passlack W., Muller S., Hanisch F.G., Franz T., Li X. (2014). Secretome profiling of primary human skeletal muscle cells. Biochim. Biophys. Acta.

[61] Le Bihan M.C., Bigot A., Jensen S.S., Dennis J.L., Rogowska-Wrzesinska A., Laine J., Gache V., Furling D., Jensen O.N., Voit T. (2012). In-depth analysis of the secretome identifies three major independent secretory pathways in differentiating human myoblasts. J. Proteom..

[62] Henningsen J., Pedersen B.K., Kratchmarova I. (2011). Quantitative analysis of the secretion of the MCP family of chemokines by muscle cells. Mol. Biosyst..

[63] Graham M.F., Willey A., Adams J., Yager D., Diegelmann R.F. (1996). Interleukin 1 beta down-regulates collagen and augments collagenase expression in human intestinal smooth muscle cells. Gastroenterology.

[64] Huang M., Sharma S., Zhu L.X., Keane M.P., Luo J., Zhang L., Burdick M.D., Lin Y.Q., Dohadwala M., Gardner B. (2002). IL-7 inhibits fibroblast TGF-beta production and signaling in pulmonary fibrosis. J. Clin. Investig..

[65] Reitamo S., Remitz A., Tamai K., Uitto J. (1994). Interleukin-10 modulates type I collagen and matrix metalloprotease gene expression in cultured human skin fibroblasts. J. Clin. Investig..

[66] Dufour A.M., Alvarez M., Russo B., Chizzolini C. (2018). Interleukin-6 and Type-I Collagen Production by Systemic Sclerosis Fibroblasts Are Differentially Regulated by Interleukin-17A in the Presence of Transforming Growth Factor-Beta 1. Front. Immunol..

[67] Aoudjehane L., Pissaia A., Scatton O., Podevin P., Massault P.P., Chouzenoux S., Soubrane O., Calmus Y., Conti F. (2008). Interleukin-4 induces the activation and collagen production of cultured human intrahepatic fibroblasts via the STAT-6 pathway. Lab. Investig..

[68] Kim Y.M., Park S.K., Xu J., Yeon S.H. (2017). Role of IL-25 in Extracellular Matrix and Collagen Production in Nasal Fibroblast. J. Allergy Clin. Immun..

[69] Franze E., Dinallo V., Laudisi F., Colantoni A., Ortenzi A., Giuffrida P., Di Carlo S., Sica G., Di Sabatino A., Monteleone G. (2020). Interleukin-34 Stimulates Gut Fibroblasts to Produce Collagen Synthesis. Digest. Liver Dis..

[70] Dong K., Markova N., Smiles K., Yarosh D. (2008). TNF-alpha and IL-6 regulate collagen 1 and MMP-1 in dermal fibroblasts. Bicyclic monoterpene diols suppress MMP-1 secretion and increase collagen production through TNF-alpha signaling. J. Am. Acad. Dermatol..

[71] Best K.T., Lee F.K., Knapp E., Awad H.A., Loiselle A.E. (2019). Deletion of NFKB1 enhances canonical NF-kappaB signaling and increases macrophage and myofibroblast content during tendon healing. Sci. Rep..

[72] Millar N.L., Murrell G.A.C., McInnes I.B. (2017). Inflammatory mechanisms in tendinopathy—Towards translation. Nat. Rev. Rheumatol..

[73] Xie S.T., Li J., Wang J.H., Wu Q., Yang P., Hsu H.C., Smythies L.E., Mountz J.D. (2010). IL-17 Activates the Canonical NF-kappa B Signaling Pathway in Autoimmune B Cells of BXD2 Mice To Upregulate the Expression of Regulators of G-Protein Signaling 16. J. Immunol..

[74] Li J., Lau G.K.K., Chen L.L., Dong S.S., Lan H.Y., Huang X.R., Li Y., Luk J.M., Yuan Y.F., Guan X.Y. (2011). Interleukin 17A Promotes Hepatocellular Carcinoma Metastasis via NF-kB Induced Matrix Metalloproteinases 2 and 9 Expression. PLoS ONE.

[75] Best K.T., Nichols A.E.C., Knapp E., Hammert W.C., Ketonis C., Jonason J.H., Awad H.A., Loiselle A.E. (2020). NF-kappa B activation persists into the remodeling phase of tendon healing and promotes myofibroblast survival. Sci. Signal..

[76] Ryan D.G., Taliana L., Sun L.J., Wei Z.G., Masur S.K., Lavker R.M. (2003). Involvement of S100A4 in stromal fibroblasts of the regenerating cornea. Investig. Ophthalmol. Vis. Sci..

[77] Droguett R., Cabello-Verrugio C., Riquelme C., Brandan E. (2006). Extracellular proteoglycans modify TGF-beta bio-availability attenuating its signaling during skeletal muscle differentiation. Matrix Biol..

[78] El-Akkawi A.I., Joanroy R., Barfod K.W., Kallemose T., Kristensen S.S., Viberg B. (2018). Effect of Early Versus Late Weightbearing in Conservatively Treated Acute Achilles Tendon Rupture: A Meta-Analysis. J. Foot Ankle Surg..

[79] Huang J.Z., Wang C., Ma X., Wang X., Zhang C., Chen L. (2015). Rehabilitation Regimen After Surgical Treatment of Acute Achilles Tendon Ruptures A Systematic Review With Meta-analysis. Am. J. Sport Med..

[80] Valkering K.P., Aufwerber S., Ranuccio F., Lunini E., Edman G., Ackermann P.W. (2017). Functional weight-bearing mobilization after Achilles tendon rupture enhances early healing response: A single-blinded randomized controlled trial. Knee Surg. Sport Tr A.

[81] Niehoff A., Kersting U.G., Helling S., Dargel J., Maurer J., Thevis M., Bruggemann G.P. (2010). Different mechanical loading protocols influence serum cartilage oligomeric matrix protein levels in young healthy humans. Eur. J. Appl. Physiol..

[82] Luc-Harkey B.A., Franz J.R., Hackney A.C., Blackburn J.T., Padua D.A., Pietrosimone B. (2018). Lesser lower extremity mechanical loading associates with a greater increase in serum cartilage oligomeric matrix protein following walking in individuals with anterior cruciate ligament reconstruction. Clin. Biomech..

[83] El-Habta R., Andersson G., Kingham P.J., Backman L.J. (2021). Anti-apoptotic effect of adipose tissue-derived stromal vascular fraction in denervated rat muscle. Stem Cell Res. Ther..

